# Supplementing cultured human myotubes with hibernating bear serum results in increased protein content by modulating Akt/FOXO3a signaling

**DOI:** 10.1371/journal.pone.0263085

**Published:** 2022-01-25

**Authors:** Mitsunori Miyazaki, Michito Shimozuru, Toshio Tsubota

**Affiliations:** 1 Department of Integrative Physiology, Graduate School of Biomedical and Health Sciences, Hiroshima University, Hiroshima, Japan; 2 Department of Physical Therapy, School of Rehabilitation Sciences, Health Sciences University of Hokkaido, Hokkaido, Japan; 3 Faculty of Veterinary Medicine, Laboratory of Wildlife Biology and Medicine, Hokkaido University, Hokkaido, Japan; University of Minnesota Medical School, UNITED STATES

## Abstract

Hibernating bears remain in their dens for 5–7 months during winter and survive without eating or drinking while staying inactive. However, they maintain their physical functions with minimal skeletal muscle atrophy and metabolic dysfunction. In bears, resistance to skeletal muscle atrophy during hibernation is likely mediated by seasonally altered systemic factors that are independent of neuromuscular activity. To determine whether there are components in bear serum that regulate protein and energy metabolism, differentiated human skeletal muscle cells were treated with bear serum (5% in DMEM/Ham’s F-12, 24 h) collected during active summer (July) and hibernating winter (February) periods. The serum samples were collected from the same individual bears (*Ursus thibetanus japonicus*, n = 7 in each season). Total protein content in cultured skeletal muscle cells was significantly increased following a 24 h treatment with hibernating bear serum. Although the protein synthesis rate was not altered, the expression of MuRF1 protein, a muscle-specific E3 ubiquitin ligase was significantly decreased along with a concomitant activation of Akt/FOXO3a signaling. Increased levels of insulin-like growth factor-1 (IGF-1) were also observed in hibernating bear serum. These observations suggest that protein metabolism in cultured human myotubes may be altered when incubated with hibernating bear serum, with a significant increase in serum IGF-1 and diminished MuRF1 expression, a potential target of Akt/FOXO3a signaling. A protein sparing phenotype in cultured muscle cells by treatment with hibernating bear serum holds potential for the development of methods to prevent human muscle atrophy and related disorders.

## Introduction

Skeletal muscle is a highly plastic tissue in the human body. Increased contractile activity through physical exercise triggers skeletal muscle hypertrophy, transformation to a slow-twitch muscle type, and increased oxidative capacity through mitochondrial biogenesis [1,2]. By contrast, prolonged periods of physical inactivity or malnutrition result in physical and metabolic dysfunction, such as skeletal muscle atrophy, transformation to a fast-twitch muscle type, and inadequate energy metabolism [3–5]. Since the loss of skeletal muscle mass and metabolic dysfunction are directly related to shorter health span [6], sarcopenia [7], and increased morbidity of lifestyle-related diseases [5], maintaining skeletal muscle mass and metabolic characteristics is an important challenge to promote healthy human life.

For hibernating animals, hibernation is a key survival strategy to survive the winter environment of food deprivation and cold temperatures by decreasing physical activity and whole-body metabolism [8,9]. Most animal species including humans suffer a significant decline in physical function with continued inactivity, whereas hibernating animals, such as bears, successfully maintain their physical function even after half a year of inactivity and fasting during hibernation. The muscle mass and strength of hibernating bears are well preserved despite 5–6 months of continued inactivity [10–13]. By contrast, even in hibernating animals, when muscle activity is restricted during the active summer period, bears show a significant decrease in skeletal muscle mass [12]. Thus, resistance to skeletal muscle atrophy and the maintenance of metabolism in hibernating bears are likely achieved by humoral or systemic factors that are induced during hibernation.

A previous study demonstrated that plasma from hibernating bears incubated with rat skeletal muscle *ex vivo* showed an inhibitory effect on the proteolytic system of skeletal muscle [14]. Another study, in which serum from hibernating bears was administered to primary cultured human skeletal muscle cells, revealed an increased protein content in the muscle cells through the inhibition of proteolysis [15]. These earlier studies suggested that serum or plasma from hibernating bears may contain some systemic circulating factor that influences the regulation of skeletal muscle protein metabolism and contributes to the maintenance of skeletal muscle mass during hibernation. In the present study, we determined whether the addition of hibernating bear serum (HBS) to cultured human skeletal muscle cells alters the protein and energy metabolism of skeletal muscle *in vitro*. Particularly, we analyzed whether the addition of HBS alters signaling pathways that regulate protein and energy metabolism in cultured human skeletal muscle cells compared with serum collected during the active phase.

## Materials and methods

### Antibodies

Phospho-S6K1 (Thr389, Cat#: 9205), phospho-S6K1 (Thr421/Ser424, Cat#: 9204), phospho-S6 ribosomal protein (Ser235/236, Cat#: 4858), phospho-S6 ribosomal protein (Ser240/244, Cat#: 5364), S6 ribosomal protein (Cat#: 2317), phospho-Akt (Thr308, Cat#: 5106), phospho-Akt (Ser473, Cat#: 4060), pan-Akt (Cat#: 4691), phospho-FoxO3a (Ser253, Cat#: 9466), phospho-FoxO3a (Ser318/321, Cat#: 9465), FoxO3a (Cat#: 2497S) were obtained from Cell Signaling Technology (Danvers, MA, USA). GAPDH (Cat#: sc-32233), Lamin A/C (Cat#: sc-7292), and S6K1 (Cat#: sc-230) were from Santa Cruz Biotechnology (Santa Cruz, CA, USA). LC3 (Cat#: M186-3) and p62 (Cat#: PM045) were from MBL (Nagoya, Aichi, Japan). Puromycin antibody (Cat#: MABE343) was from EMD Millipore (Merck, Darmstadt, Germany). Anti-Fbx32 (Cat#: ab168372), anti-MURF1 (Cat#: ab172479), and total oxphos human wb antibody cocktail (Cat#: ab110411) were from Abcam (Cambridge, MA, USA). IRDye 800CW Goat anti-Mouse IgG (Cat#: 926–32210) and IRDye 680LT Goat anti-Rabbit IgG (Cat#: 926–68021) were from LI-COR Biosciences (Lincoln, NE, USA). Alexa Fluor 680-conjugated Goat anti-Mouse IgG2a (Cat#: A31563) and Alexa Fluor 594-conjugated Goat anti-Mouse IgG2b (Cat#: A21145) were from Thermo Fisher Scientific (Rockford, IL, USA). The hybridoma (MF 20) developed by Fischman, D.A. was obtained from the Developmental Studies Hybridoma Bank, created by the NICHD of the NIH and maintained at The University of Iowa, Department of Biology, Iowa City, IA 52242.

### Animal care and use

All experimental procedures and animal care performed in this study were conducted according to Institutional Guidelines for Animal Care and Use and approved by the Animal Care and Use Committee of the Graduate School of Veterinary Medicine, Hokkaido University (Permit Numbers: Vet17006 and Vet18-0179). A total of seven nonpregnant, female Japanese black bears (*Ursus thibetanus japonicus*), between 5 and 15 years of age, housed in Ani Mataginosato Bear Park (Akita Prefecture, Japan, N40° E140.4°) were used for this study. All animal care and handling procedures were followed as previously described [10,16]. Briefly, animals were fed with dried corn (360 kcal/100 g, approximately 1.5 kg/head) combined with fruits and vegetables as supplements once a day at 16:00 h during the active period (i.e., from late April to late November). Two weeks before or after the fasting period (i.e., late November/early December to early/mid-April) as a transition phase to and from torpor status, the amount of feeding was reduced to one-third (0.5 kg cornmeal/head) compared with the active period. All animals were kept isolated in the indoor dark rooms for denning and had no access to food during the torpor period. Access to drinking water was allowed *ad libitum* throughout the year. ***Serum sample collection***: During the 2017–2018 season, blood samples were collected from each of the same individual bear under anesthesia in mid-July (normal activity period) and in late February (hibernation period). Blood samples were incubated at room temperature (approximately 24°C in a temperature-controlled room) for 60 min to clot and then centrifuged for 10 min at 1,042×g. The serum supernatants were collected, quickly frozen in liquid nitrogen, and stored at −80°C until use. Animals were anesthetized with an intramuscular administration of a 3.0 mg/kg zolazepam hydrochloride and tiletamine hydrochloride cocktail and 40 μg/kg medetomidine hydrochloride using a blow dart shot. Feeding was restricted overnight (approximately 15–16 h) until the collection of anesthesia and serum was completed the following morning. Meloxicam (subcutaneously at 0.2 mg/kg for analgesia) and atipamezole hydrochloride (intramuscularly at 200 μg/kg as an antagonist to medetomidine hydrochloride) were administered to aid recovery.

### Cell culture

All cell culture experiments were performed in a humidified environment at 37°C in a 5% CO_2_ atmosphere. Human skeletal muscle myoblasts (HSMMs, Cat#: CC-2580, Lot#: 29394) and culture medium (Skeletal Muscle Cell Growth Medium-2 BulletKit, Cat#: CC-3245) were purchased from Lonza (Basel, Switzerland). DMEM-F12 was obtained from FUJIFILM Wako (Osaka, Japan). HSMMs were cultured in a growth medium according to the manufacturer’s instructions until the culture reached 60%–70% confluence. To induce differentiation, the culture medium was switched to a fusion medium (DMEM-F12 supplemented with 2% horse serum). The fusion medium was replaced every other day until multinucleated myotubes were observed throughout the culture. ***Bear serum treatment***: Cultured myotubes at 5 days of differentiation were treated with bear serum (5% in DMEM-F12) collected during active summer (July) and hibernating winter (February) periods for 24 h.

### Immunocytochemistry

Cultured myotubes were fixed in 4% paraformaldehyde, permeabilized with 0.1% Triton X-100, and blocked with 1% bovine serum albumin. Mouse anti-myosin heavy chain (MHC), sarcomere (MF 20) and Alexa Fluor 594-conjugated Goat anti-Mouse IgG2b antibodies were used for detecting MHC localization. Cells were mounted with Vectashield mounting medium with DAPI for nuclei counterstaining. All images were captured using the Keyence BZ9000 imaging system (Keyence, Osaka, Japan). The number of DAPI-positive signals in one visual field (20X) under the microscope was counted as the number of nuclei. Four non-overlapped areas per sample were randomly chosen and the average value was calculated.

### Measurement of protein synthesis

The rate of protein synthesis was measured using the surface sensing of translation (SUnSET) technique [17,18]. This technique uses the antibiotic puromycin as a structural analog of tyrosyl-tRNA. Briefly, cultured myotubes were treated with 5% bear serum in DMEM-F12 for 24 h, and the medium was replaced with a fresh culture medium containing puromycin (10 μg/ml) for the final 90 min. Myotubes were then collected, and the amount of puromycin incorporated into the nascent peptide chains was determined via western blot analysis using an antipuromycin antibody.

### Protein extraction and western blotting

For whole cell protein extraction, myotube samples were washed twice with phosphate-buffered saline and then scraped into ice-cold lysis buffer (1% NP-40, 0.5% sodium deoxycholate, 0.1% SDS, 50 mM NaCl, 20 mM Tris–HCl [pH, 7.6], 1 mM PMSF, 5 mM benzamidine, 1 mM EDTA, 5 mM N-ethylmaleimide, 50 mM NaF, 25 mM B-glycerophosphate, 1 mM sodium orthovanadate, and 1X protease inhibitor cocktail [Nacalai Tesque, Kyoto, Japan]). Lysed samples were then centrifuged at 16,000×g for 10 min at 4°C, and the supernatants were collected for analysis. Protein concentration was determined using the BCA Protein Assay Kit (Thermo Fisher Scientific, Rockford, IL, USA). For the assessment of FOXO3a translocation, nuclear and cytoplasmic extracts were isolated using an NE-PER Nuclear and Cytoplasmic Extraction Kit according to the manufacturer’s instructions (Thermo Fisher Scientific, Rockford, IL, USA). Protein samples were separated using a precast polyacrylamide gel system (e-PAGEL; ATTO, Tokyo, Japan) and transferred to PVDF membranes (Immobilon-FL Transfer Membrane; Merck, Darmstadt, Germany). Membranes were then blocked in Odyssey Blocking Buffer (LI-COR Biosciences, Lincoln, NE, USA) and incubated with diluted primary and secondary antibodies. Bound antibody complexes were scanned and quantified using the Odyssey CLx Imaging System operated with Image Studio Version 3.1 software (LI-COR Biosciences, Lincoln, NE, USA).

### RNA isolation and real-time PCR

Total RNA was prepared using the RNeasy Mini Kit (Qiagen, Hilden, Germany) according to the manufacturer’s instructions. The isolated RNA was quantified via spectrophotometry (λ = 260 nm). First-strand cDNA synthesis was performed using the PrimeScript RT Reagent Kit. SYBR Premix Ex Taq II and the TaKaRa Thermal Cycler Dice Real Time System TP850 (Takara Bio, Shiga, Japan) were used for the amplification and quantification of each gene. Primer sequences (5’-3’, forward and reverse, respectively) for the qPCR assays were designed on the basis of the public database, PrimerBank (https://pga.mgh.harvard.edu/primerbank/index.html) as follows: Pgc1a, GCTTTCTGGGTGGACTCAAGT and GAGGGCAATCCGTCTTCATCC; Pgc1b, GATGCCAGCGACTTTGACTC and ACCCACGTCATCTTCAGGGA; Ucp3, GGGTCAACCTGGGATGTAGC and TCCCTAACCCTCCCCATCAG; Cycs, CTTTGGGCGGAAGACAGGTC and TTATTGGCGGCTGTGTAAGAG; Cox4, CAGGGTATTTAGCCTAGTTGGC and AGACAGGTGCTTGACATGGG; Cpt1b, GCGCCCCTTGTTGGATGAT and CTGTTCACCATGAGAGGGCT; Actb, GTCATTCCAAATATGAGATGCGT and GCTATCACCTCCCCTGTGTG. The expression of each gene was determined via the 2^−ΔΔCT^ method using Actb as an internal control.

### IGF-1 ELISA assay

Levels of insulin-like growth factor-1 (IGF-1) in bear serum were determined using a human IGF-1 ELISA kit (OKCD05748, Aviva Systems Biology, San Diego, CA, USA) following the manufacturer’s instructions. This ELISA kit was originally designed for quantifying human IGF-1; however, prior confirmation was made that the IGF-1 peptide sequence in bears and humans is 100% identical.

### Statistical analysis

All results are reported as means ± standard errors. Statistical differences between the summer active period (Active) and the winter-hibernation period (Hibernation) were determined using a Student’s t-test. For all comparisons, the level of statistical significance was set at p < 0.05.

## Results

### Alteration of total protein content in cultured human myotubes: Treatment with hibernating period vs. summer active bear serum

**Fig 1:**
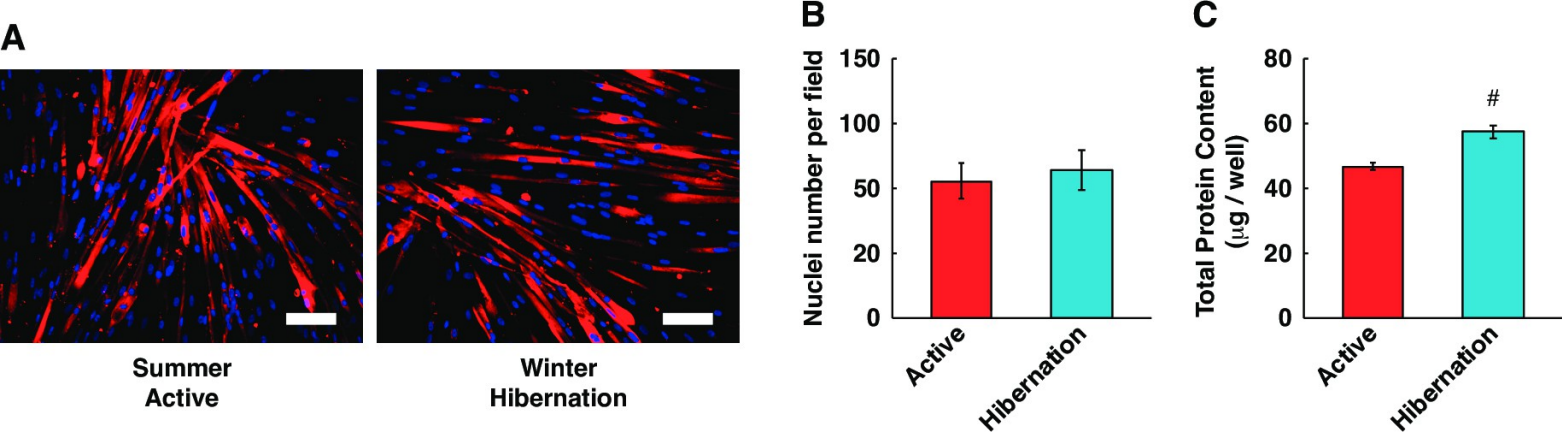
Alteration of total protein contents in cultured human skeletal muscle cells: Winter-hibernation period vs. summer-active period. (A) Typical immunostaining images of cultured human skeletal muscle cells at 5 days of differentiation following 24 h treatment with bear serum. Red, myosin heavy chain (MF20); blue, DAPI. The occasional small bright red dot is a staining artifact. The scale bar shows 100 μm. (B) The number of DAPI-positive signals per field was counted as the nuclei number. (C) The total protein contents were significantly increased in cultured myotubes following 24 h treatment with hibernating bear serum compared to the active period serum. Data are expressed as mean ± standard errors. Significant differences: #, p < 0.05.

In this study, we investigated whether serum from hibernating bears could be added to cultured human skeletal muscle cells to alters the protein metabolism of skeletal muscle *in vitro*. Although we did not observe any obvious morphological changes (Fig 1A) or increase in the number of nuclei (Fig 1B) in differentiated human myotubes following a 24 h incubation with bear serum, the total protein content was significantly increased when cultured in HBS compared with the active period bear serum (ABS) (Fig 1C).

### Regulation of protein anabolism in myotubes

**Fig 2:**
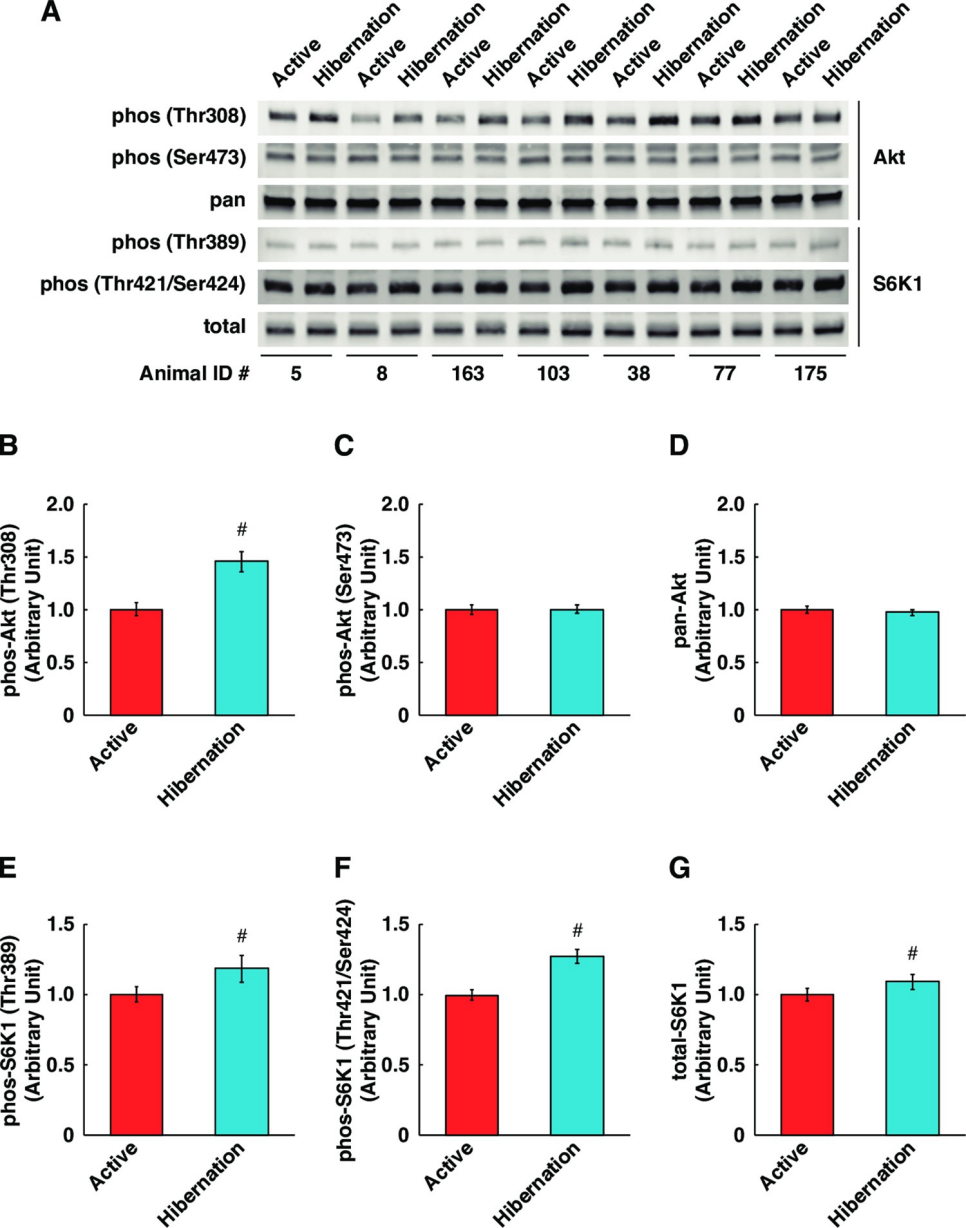
Enhanced Akt/mTORC1 signaling in cultured human skeletal muscle cells with hibernating bear serum. (A) Representative images of Western blotting for phosphorylated and total expression of Akt and S6K1. Phosphorylation status of Akt at Thr308 (B), Ser473 (C), and total Akt expression (D) were quantified. Phosphorylation status of S6K1 at Thr389 (E), Thr421/Ser424 (F), and total S6K1 expression (G) were quantified. The Animal ID indicates the identification number of each individual bear. The experimental group for each season was consisted of identical bear individuals. N = 7 in each group. Data are expressed as mean ± standard errors. Significant differences: #, p < 0.05.

**Fig 3:**
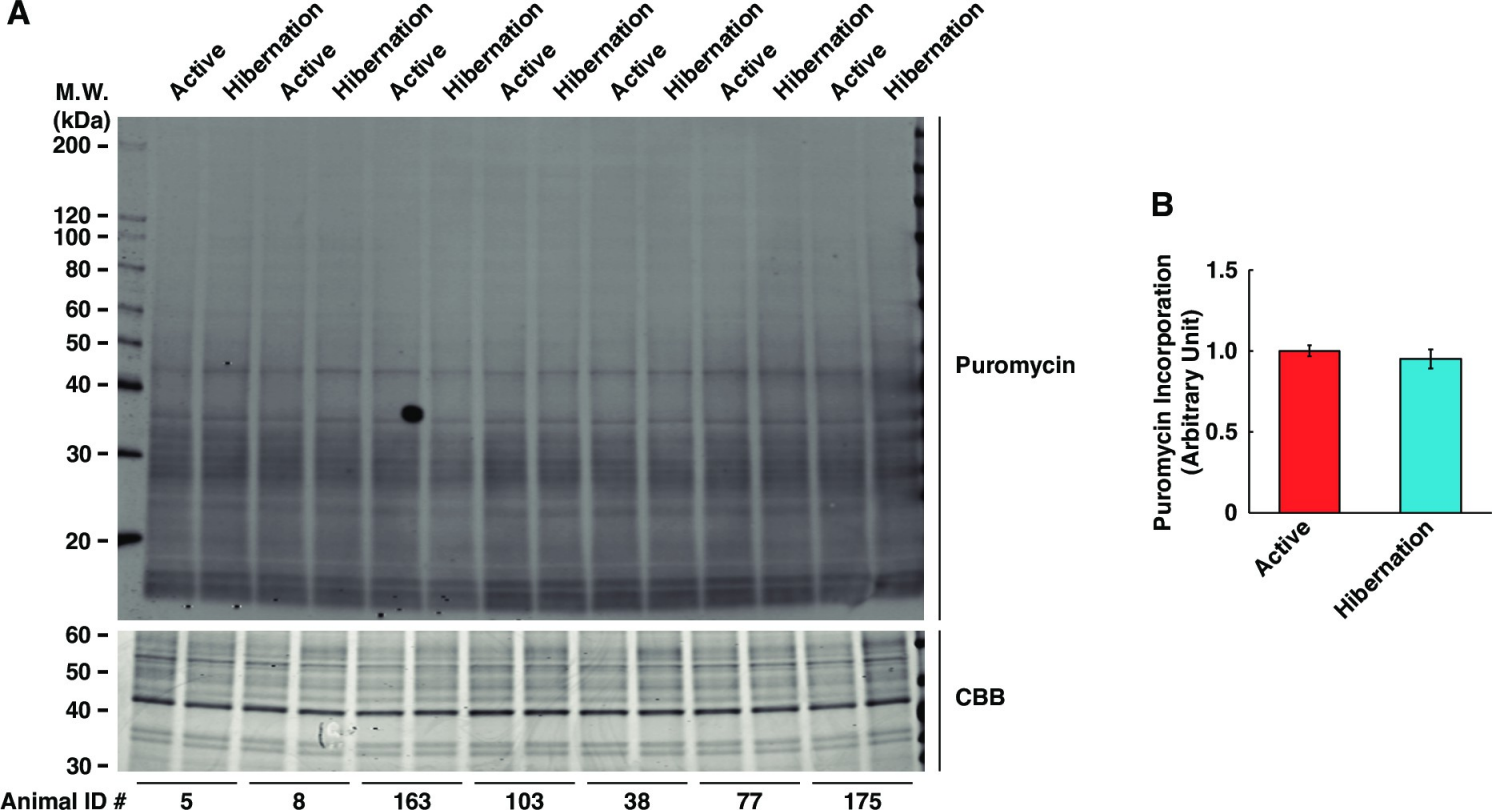
No alteration of protein anabolisms in cultured human skeletal muscle cells with hibernating bear serum. (A) Representative images of Western blotting with antipuromycin antibody. Equal amounts of protein loading in each sample were confirmed by the staining with Coomassie Brilliant Blue. (B) Protein synthesis in cultured myotubes was determined by quantification (normalized to summer-active group) of the incorporated puromycin. The Animal ID indicates the identification number of each individual bear. The experimental group for each season was consisted of identical bear individuals. N = 7 in each group. Data are expressed as mean ± standard errors.

Since fully differentiated myotubes were used in this study, alterations of cellular protein content essentially reflected the balance of protein synthesis and degradation. Given that HBS increased total protein content, we next examined the activation status of the Akt and mechanistic target of rapamycin complex 1 (mTORC1) pathways, which have been implicated as central regulators of protein synthesis in skeletal muscle. The phosphorylation of Akt at Thr308, an indicator of Akt kinase activity, was significantly increased in cultured myotubes following treatment with HBS compared with ABS (Fig 2B). Additionally, S6K1 phosphorylation, a direct downstream target of mTORC1 activity, was also significantly enhanced both at Thr389 (Fig 2E) and Thr421/Ser424 (Fig 2F) sites. Total protein expression of S6K1 was also increased (Fig 2G). The Akt phosphorylation at Ser473 and expression level of pan-Akt were not altered (Fig 2C and 2D). We examined the rate of protein synthesis, but no significant alterations were observed (Fig 3A and 3B). These results suggest that although treatment with HBS induces activation of the Akt/mTORC1 pathway, this does not directly induce the activation of protein synthesis in cultured human skeletal muscle cells.

### Regulation of protein catabolism in human myotubes

**Fig 4:**
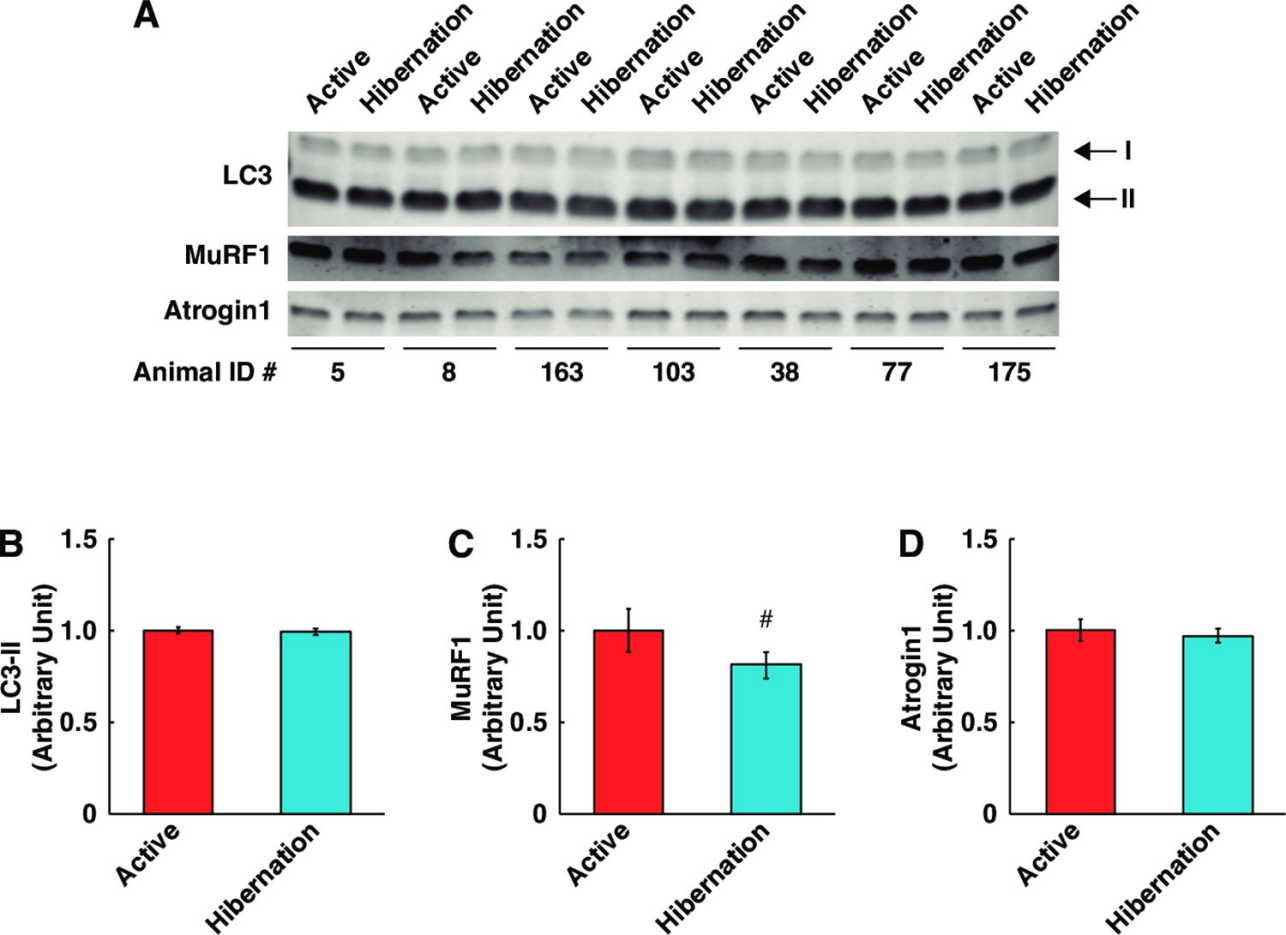
Diminished protein expression of proteolysis system in cultured human skeletal muscle cells with hibernating bear serum. (A) Representative images of Western blotting for each protein. Protein expression levels of LC3 (B), MuRF1 (C) and Atrogin1 (D) were quantified. The Animal ID indicates the identification number of each individual bear. The experimental group for each season was consisted of identical bear individuals. N = 7 in each group. Data are expressed as mean ± standard errors. Significant differences: #, p < 0.05.

Because total protein content increased while the protein synthesis system was not affected, we next examined the effect of HBS on the proteolytic system in cultured myotubes. Two major proteolytic pathways occur in skeletal muscle: the autophagy–lysosome system and the ubiquitin–proteasome system. Microtubule-associated protein 1 light chain 3 (LC3)-II, a marker for autophagosome formation, was not altered; suggesting that autophagic flux in cultured myotubes is unchanged (Fig 4B). By contrast, the expression of muscle RING-finger protein-1 (MuRF1) protein, a skeletal muscle-specific E3 ubiquitin ligase and known marker of muscle atrophy, was significantly decreased following HBS treatment (Fig 4C). Atrogin1 protein expression was not altered (Fig 4D). These results suggest that protein degradation through the ubiquitin–proteasome-dependent system was inhibited in human skeletal muscle cells in response to HBS treatment.

### The Akt-FOXO axis is enhanced using HBS treatment

**Fig 5:**
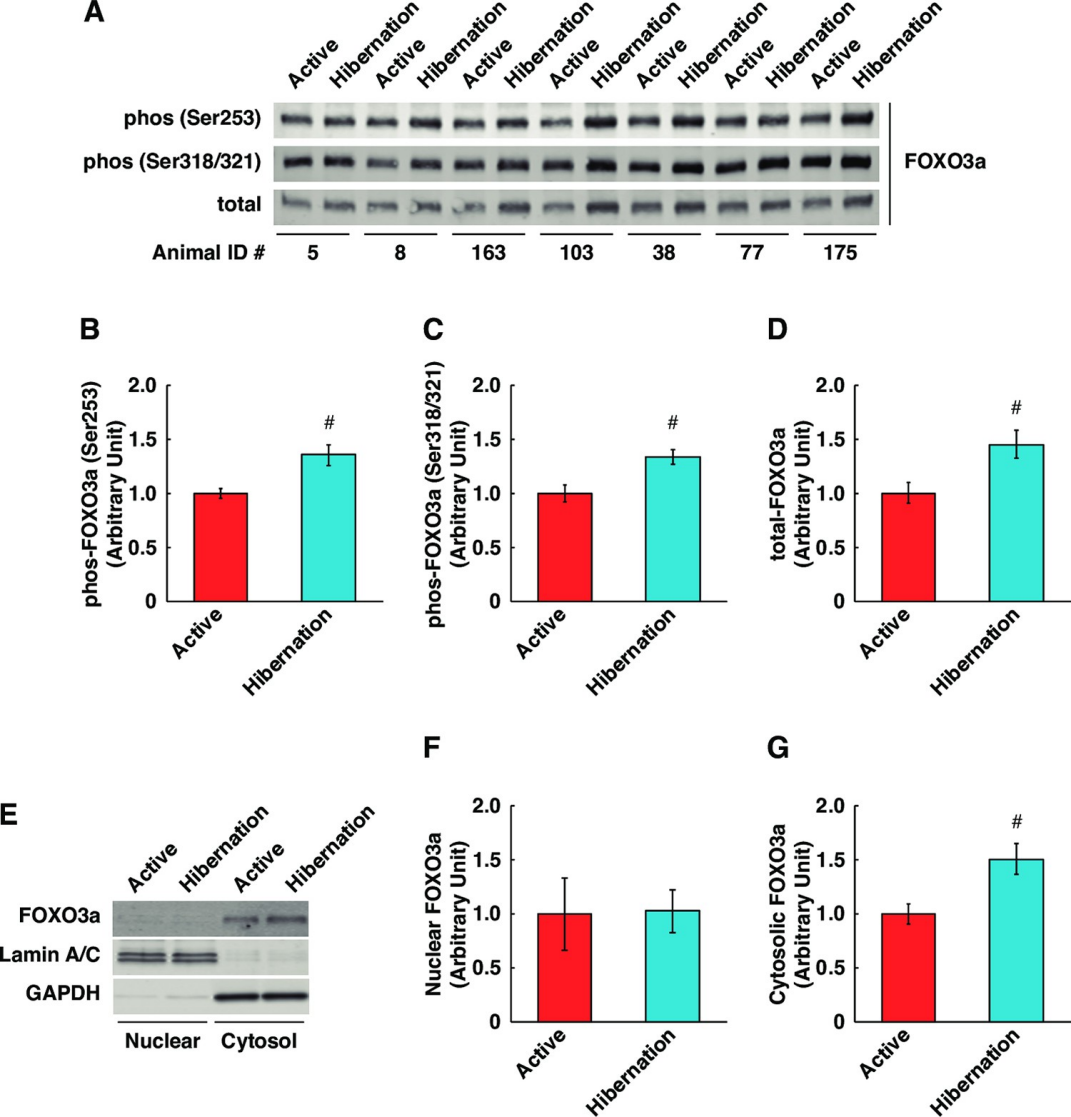
Increased phosphorylation and cytosolic-anchoring of FOXO3a in cultured human skeletal muscle cells with hibernating bear serum. (A) Representative images of Western blotting for FOXO3a with using whole cell lysates. The Animal ID indicates the identification number of each individual bear. Phosphorylation status of FOXO3a at Ser253 (B), Ser318/321 (C), and total FOXO3a expression (D) were quantified. (E) Representative images of Western blotting for FOXO3a with using subcellular fraction. Lamin A/C was used as marker for nuclear fraction. GAPDH was used as marker for cytosolic fraction. Nuclear (F) and cytosolic (G) FOXO3a were quantified. The experimental group for each season was consisted of identical bear individuals. N = 7 in each group. Data are expressed as mean ± standard errors. Significant differences: #, p < 0.05.

The Akt-FOXO axis may represent a potential regulatory mechanism of the ubiquitin–proteasome system in skeletal muscle cells, especially by modulating the transcriptional activation of the atrogin1 and murf1 genes. The phosphorylation of FOXO3a at both Ser253 and Ser318/321 sites, which are phosphorylated by Akt directly and indirectly, are important for nuclear export to the cytosol and inhibition of FOXO3a transcription factor activity. In cultured myotubes, the phosphorylation and total expression of FOXO3a were significantly increased following HBS treatment compared with ABS (Fig 5B–5D). Additionally, subcellular fractionation of the cultured myotubes further confirmed that cytosolic FOXO3a was significantly increased following HBS treatment, while nuclear FOXO3a was unchanged (Fig 5E–5G). These results suggest, although not direct evidence, that HBS promotes the increased phosphorylation and sequestration of FOXO3a protein in the cytosolic fraction, which may lead to decreased transcriptional activity of FOXO3a.

### Alterations of IGF-1 concentration in bear serum during hibernation

**Fig 6:**
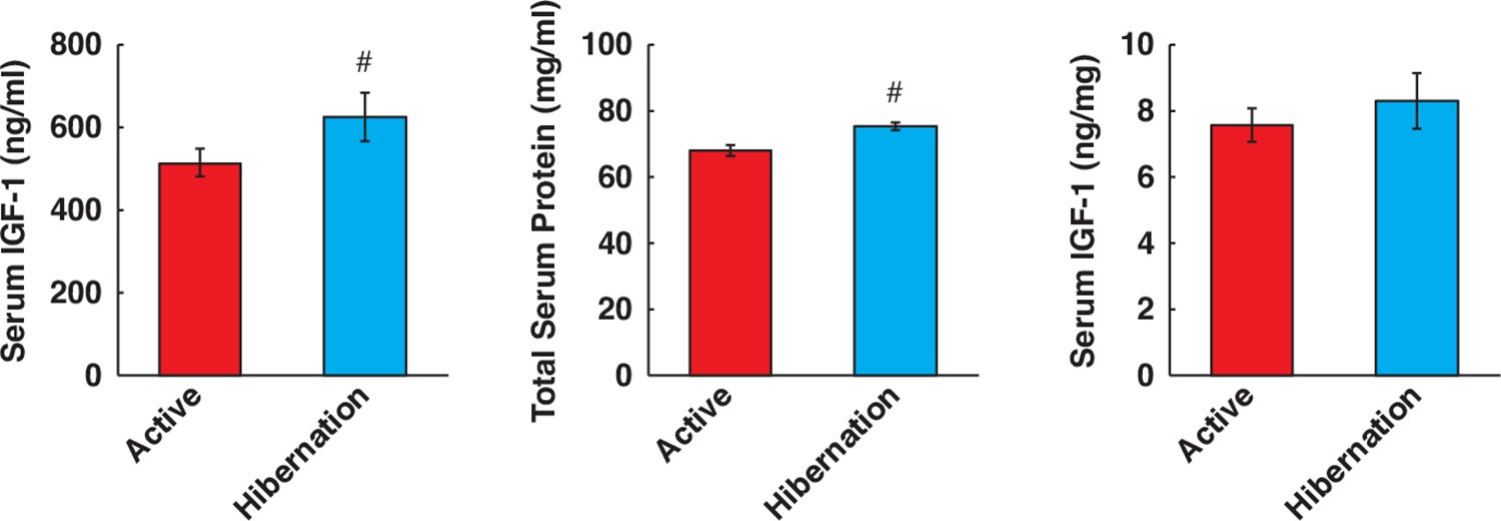
IGF-1 concentration and total protein contents in bear serum. (A) IGF-1 levels in bear serum were quantified by ELISA assay. (B) Total protein levels in bear serum were quantified. (C) IGF-1 levels were corrected with the total protein content in bear serum. The experimental group for each season was consisted of identical bear individuals. N = 7 in each group. Data are expressed as mean ± standard errors. Significant differences: #, p < 0.05.

IGF-1, a serum growth factor, is a candidate upstream factor that induces activation of the Akt-FOXO3a axis in skeletal muscle. The serum levels of IGF-1 were measured using ELISA, and we confirmed that IGF-1 content was significantly higher during hibernation compared with the active periods (Fig 6A). This result suggests that increased amounts of serum IGF-1 during hibernation affect the Akt-FOXO3a pathway in skeletal muscle cells, leading to decreased MuRF1 expression, a potential target of FOXO3a transcriptional activity. Notably, a significant increase in total protein content in the serum from hibernating bears was also observed (Fig 6B), whereas no significant increase in IGF-1 levels was detected when normalized to total protein content (Fig 6C). Thus, it can be considered that the serum components used for cell culture were simply concentrated because the water content in the serum decreased from some other causes, such as dehydration.

### Alterations of mitochondria-related proteins

**Fig 7:**
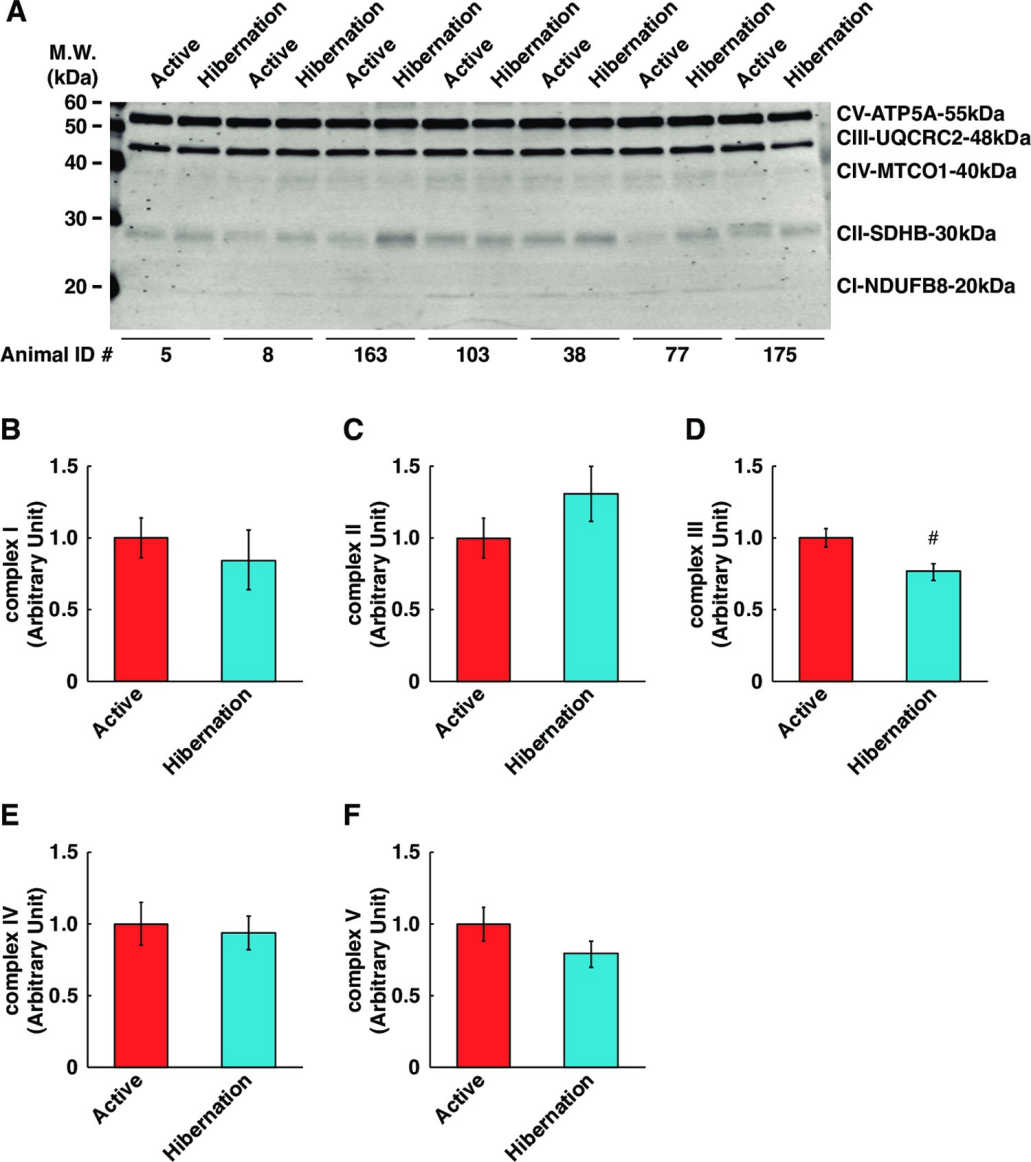
Expression levels of OXPHOS subunit proteins in cultured human skeletal muscle cells following hibernating bear serum treatment. (A) Representative images of Western blotting for total oxphos human wb antibody cocktail with using whole cell lysates. The Animal ID indicates the identification number of each individual bear. Protein content of mitochondria-related proteins in cultured myotubes were quantified. (B) complex I, (C) complex II, (D) complex III, (E) complex IV, and (F) complex V. The experimental group for each season was consisted of identical bear individuals. N = 7 in each group. Data are expressed as mean ± standard errors. Significant differences: #, p < 0.05.

**Fig 8:**
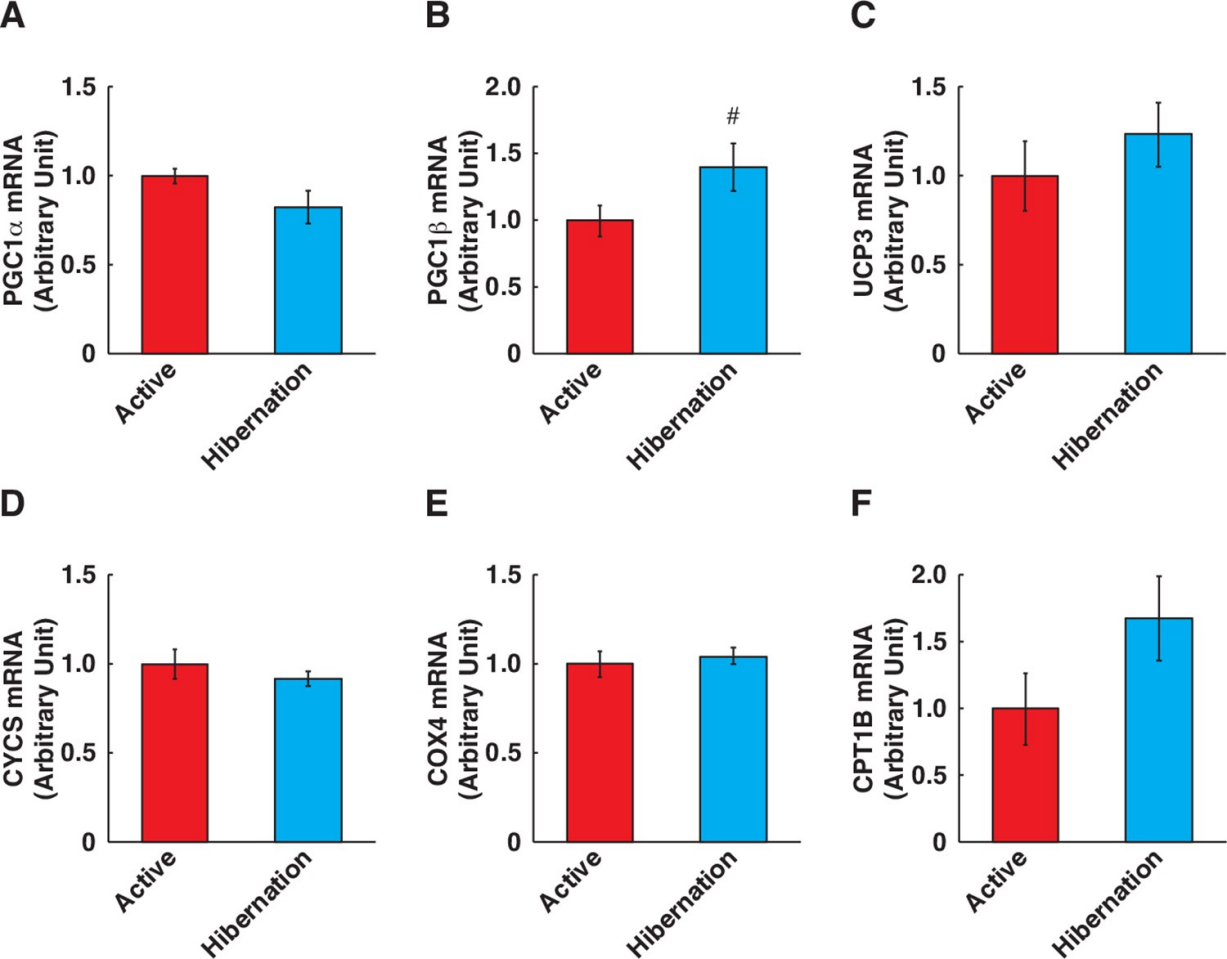
Gene expression levels of mitochondrial regulatory factors in cultured human skeletal muscle cells following hibernating bear serum treatment. (A) Gene expressions of mitochondrial regulatory factors were determined (A-F). (A) pgc1α. (B) pgc1β. (C) ucp3. (D) cycs. (E) cox4. (F) cpt1β. Expression levels of each studied gene were determined by the 2^–ΔΔCT^ method while referencing ACTB as an internal control. Absolute Ct values of ACTB PCR amplification for each sample were stable (between 15 to 16 cycles). The experimental group for each season was consisted of identical bear individuals. N = 7 in each group. Data are expressed as mean ± standard errors. Significant differences: #, p < 0.05.

Since previous studies indicated that mitochondrial biogenesis in skeletal muscle is enhanced during hibernation [10,19–21], we examined the expression of OXPHOS subunit proteins in cultured myotubes following HBS treatment. We found that there were no statistically significant alterations in most of the factors (Fig 7B–7F) and the expression of complex III was somewhat decreased following HBS treatment (Fig 7D). Also, the gene expression levels of mitochondrial biogenesis regulators (peroxisome proliferator-activated receptor gamma coactivator 1-alpha: Pgc1a, Fig 8A) and other mitochondria-related factors (mitochondrial uncoupling protein 3, cytochrome c, cytochrome c oxidase subunit 4, and carnitine palmitoyltransferase-1b, Fig 8B–8F) were not significantly altered, except for an increase in Pgc1b expression (Fig 8B). Hence, the mitochondrial biogenesis in cultured human skeletal muscle cells is not likely induced by HBS treatment, at least under these culture conditions.

## Discussion

Although the underlying mechanisms remain unknown, it has long been recognized that the skeletal muscles of hibernating animals are very well preserved or show minimal atrophy throughout the hibernation period [10–12,22–25]. Particularly in bears, it has been suggested that resistance to skeletal muscle atrophy during hibernation is likely mediated by seasonally altered physiological factors that are independent of neuromuscular activity [12]. Thus, in this study, HBS was added directly to human cultured skeletal muscle cells to determine the effects of systemic humoral factors on the regulation of protein metabolism. The experimental findings revealed that serum from hibernating black bears positively regulates muscle protein metabolism, which resulted in the accumulation of total protein content, likely through the inhibition of the protein degradation system mediated via Akt-FOXO3a signaling. This finding is consistent with that of previous studies showing that the addition of the serum from free-ranging sub-adult brown bears during hibernation inhibits the proteolysis of cultured myotubes [15], or antiproteolytic effects of plasma from hibernating brown bears using *ex vivo* treated muscle tissues [14]. Furthermore, in this study, IGF-1 content was increased in the serum from hibernating bears, which may induce the activation of the Akt-FOXO3a pathway in skeletal muscle cells. Although serum IGF-1 levels were not measured in previous reports, the activation of the FOXO pathway was observed in cultured muscle cells treated with HBS [15] or in the skeletal muscle tissue of hibernating ground squirrels [25,26]. Our findings support these earlier observations. However, it should be noted that the decrease in MuRF1 expression with HBS treatment was observed at the protein level in this study. It has been reported that MuRF1 is also controlled by post-translational modifications [27], and it is quite possible that the decrease in protein expression is not the result of repression of transcriptional activity by Akt-FOXO3a. In fact, our experimental results showed no change in the amount of FOXO3a in the nucleus, while the amount of phosphorylation and cytosolic fraction were increased. This point needs to be further investigated in the future study.

Hibernating animals also exhibit unique adaptations regarding the fiber types and metabolic properties of their skeletal muscle. In nonhibernating animals, prolonged periods of inactivity results in a shift to a faster muscle type isoform and an increase in glycolytic metabolism (decreased capacity for fat oxidation) [28,29]. In hibernating animals, however, despite prolonged inactivity, a shift to slower isoforms, an increase in oxidative metabolism, and upregulated muscle mitochondrial capacity during hibernation are evident [10,30–35]. Accordingly, we examined the effect of HBS supplementation on the expression of OXPHOS subunit proteins and mitochondria-related factors in cultured skeletal muscle cells; however, there was no significant alteration; rather, a significant decrease in the expression level of complex III was observed (Fig 7D), at least under the current experimental conditions. These earlier observations and our results suggest that the enhancement of mitochondrial biogenesis and oxidative metabolism in the skeletal muscle of hibernating animals is not affected by humoral factors but may be mediated by other factors, including neuromuscular regulation. This is consistent with long-established evidence that motor neuron innervation is a key determinant of the fiber type and metabolic properties of skeletal muscle [36,37]. Since energy production through mitochondrial respiration during hibernation is generally decreased with the suppression of systemic energy metabolism [38], it is reasonable to expect that there is no alteration of mitochondrial biogenesis in skeletal muscle cells. Notably, a recent report using the proteome analysis of hibernating bear skeletal muscle showed that the expression levels of mitochondria-related factors, including the respiratory chain complex, were generally suppressed, whereas factors involved in glycolysis and glycogenesis were preserved. This suggests that an adaptive system to retain energy substrates, such as glycogen in the muscle, may be functioning [39].

Our results indicated that the expression of Pgc1b mRNA, a regulator of mitochondrial biogenesis and energy metabolism [40,41], was increased following HBS treatment (Fig 8B). Since Pgc1b expression has also been shown to contribute to the maintenance of skeletal muscle mass by inhibiting muscle protein degradation [42], the HBS-induced alteration of the Pgc1b expression may affect the changes in muscle protein content.

An unresolved question from our study is which components in HBS influence skeletal muscle protein metabolism besides IGF-1. Although increased IGF-1 concentrations were observed in this study, this was found to be nullified following the normalization of total protein content in the bear serum. Previous studies have reported that IGF-1 concentrations in bear serum show seasonal variation, being highest during the active summer period and lowest in early hibernation and then increased again near the end of hibernation [43]. Therefore, as the present results show, it is difficult to determine whether serum IGF-1 acts to maintain protein content in cultured skeletal muscle cells.

In recent years, the exploration of novel factors in the serum or tissues of hibernating bears, which could contribute to the metabolic characteristics using omics approaches, has been extensively conducted. Lipidomic analysis of the serum from hibernating bears indicated that endogenous cannabinoid systems are specifically altered during hibernation, thereby contributing to the regulation of energy metabolism in adipose and skeletal muscle [44]. In a combined study involving miRNA sequencing and transcriptome analysis, the expression of three circulating microRNAs specifically induced during hibernation contributes to the prevention of venous thromboembolism under prolonged inactivity during hibernation [45]. The identification of circulating factors in bears during hibernation that directly contribute to the suppression of skeletal muscle proteolysis may lead to the development of powerful tools to prevent skeletal muscle atrophy associated with long-term inactivity.

## Conclusion

The total protein content of cultured human skeletal muscle cells was significantly increased following a 24 h treatment with HBS. This alteration in total protein levels was likely the result of proteolysis inhibition via HBS treatment through the suppression of MuRF1 expression, which is mediated by the activation of the Akt/FOXO3a axis. The identification of key factors in HBS that contribute to the inhibition of the proteolytic machinery may provide new approaches to prevent skeletal muscle atrophy and weakness in humans.

## Supporting information

S1 Raw images (TIF)

S2 Raw images (TIF)

S3 Raw images (TIF)

S4 Raw images (TIF)

S5 Raw images (TIF)

S6 Raw images (TIF)

S7 Raw images (TIF)
