# Supplementary figures and images for: Supplementing cultured human myotubes with hibernating bear serum results in increased protein content by modulating Akt/FOXO3a signaling
Source: PLoS One. 2022 Jan 25;17(1):e0263085. doi: 10.1371/journal.pone.0263085 (PMC8789107; doi:10.1371/journal.pone.0263085)

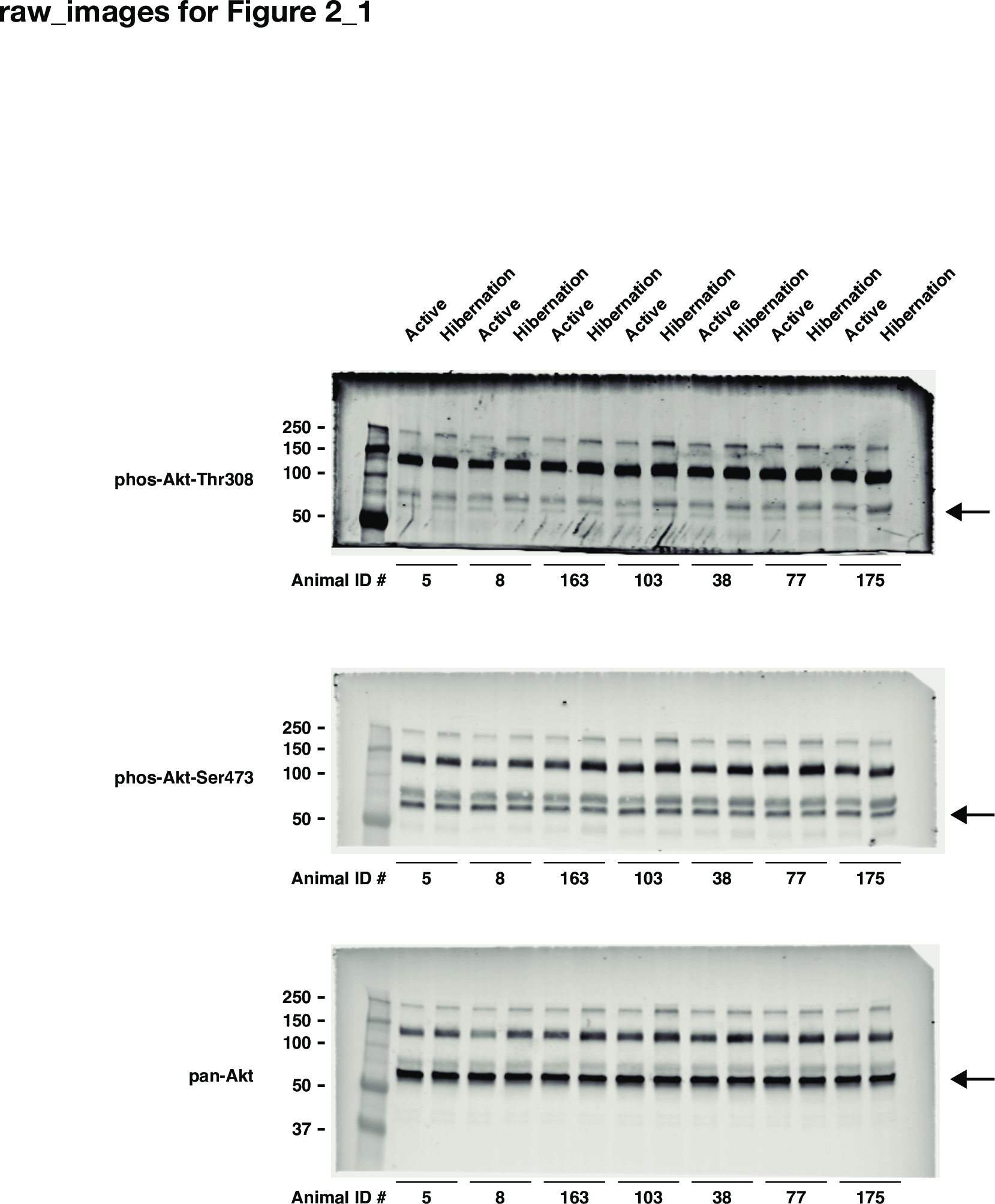

Supplement: S1 Raw images — (TIF) [file pone.0263085.s001.tif]

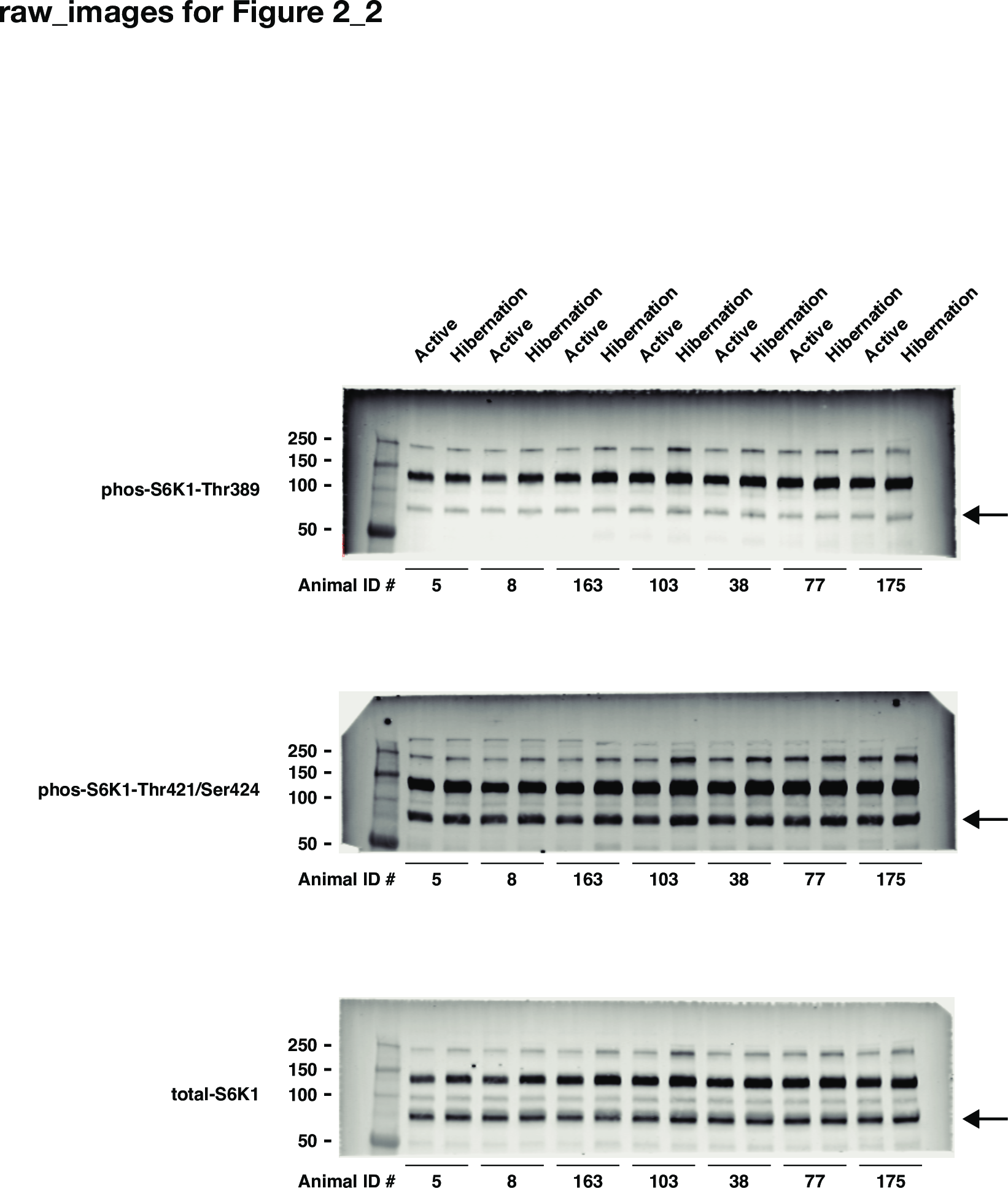

Supplement: S2 Raw images — (TIF) [file pone.0263085.s002.tif]

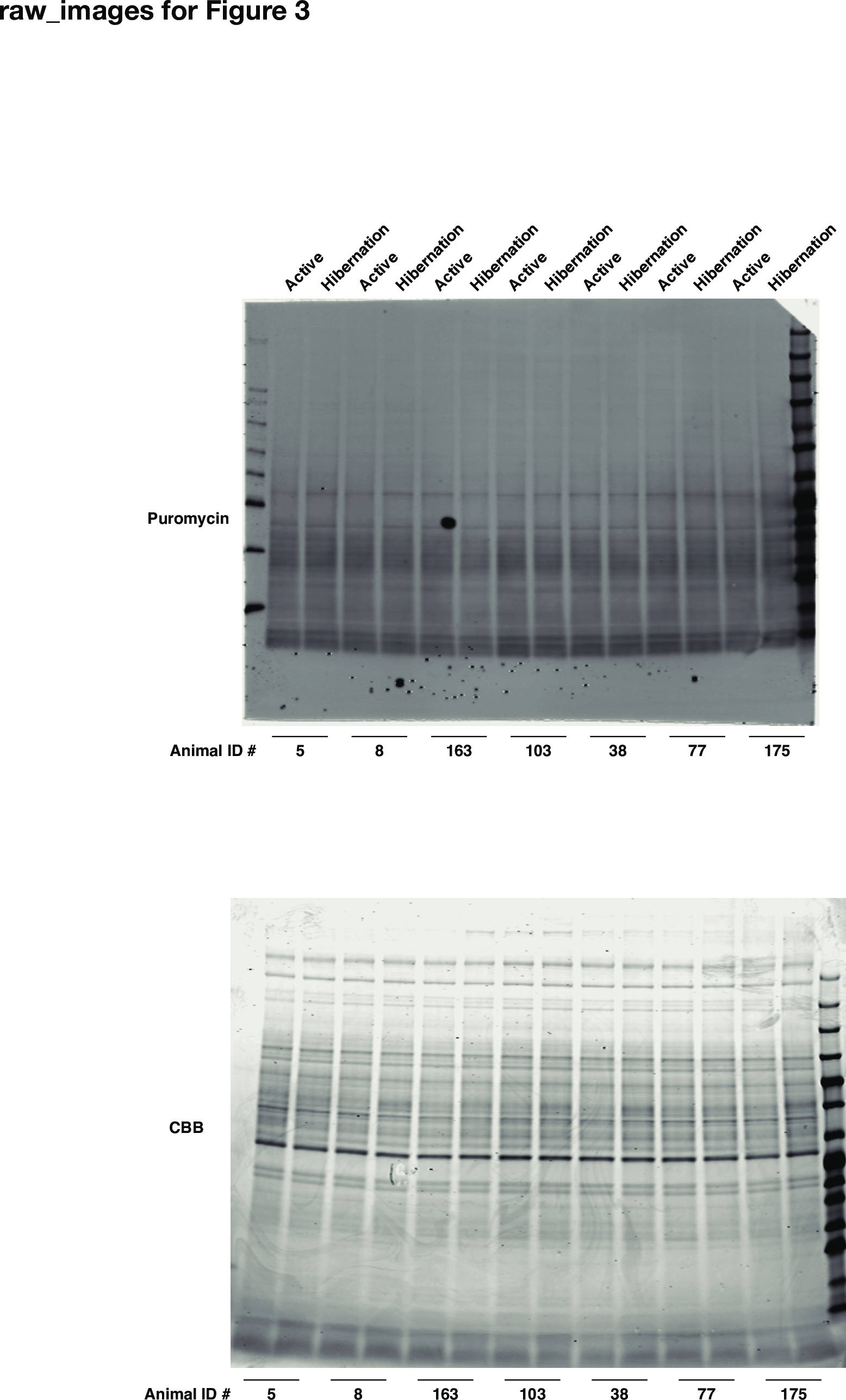

Supplement: S3 Raw images — (TIF) [file pone.0263085.s003.tif]

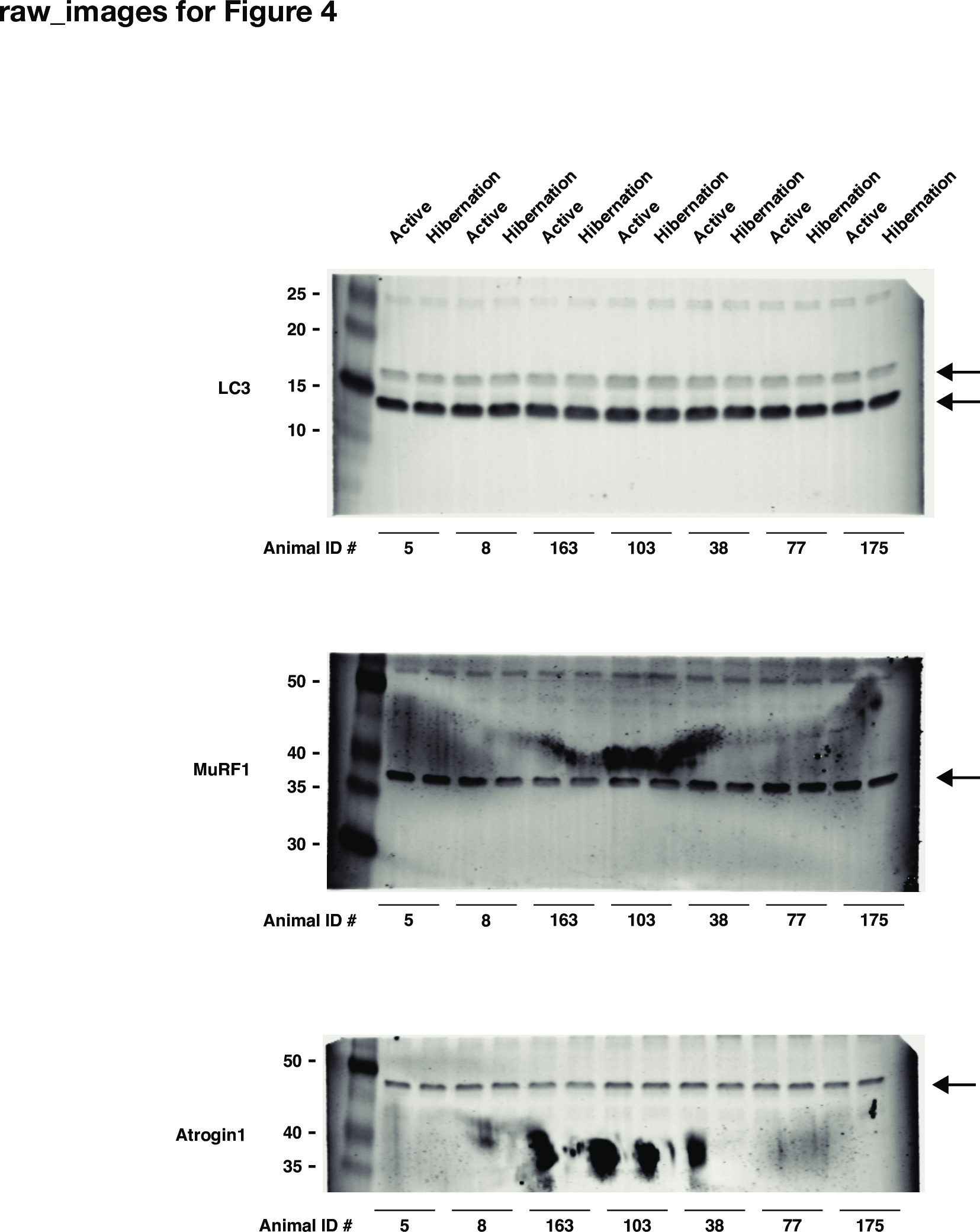

Supplement: S4 Raw images — (TIF) [file pone.0263085.s004.tif]

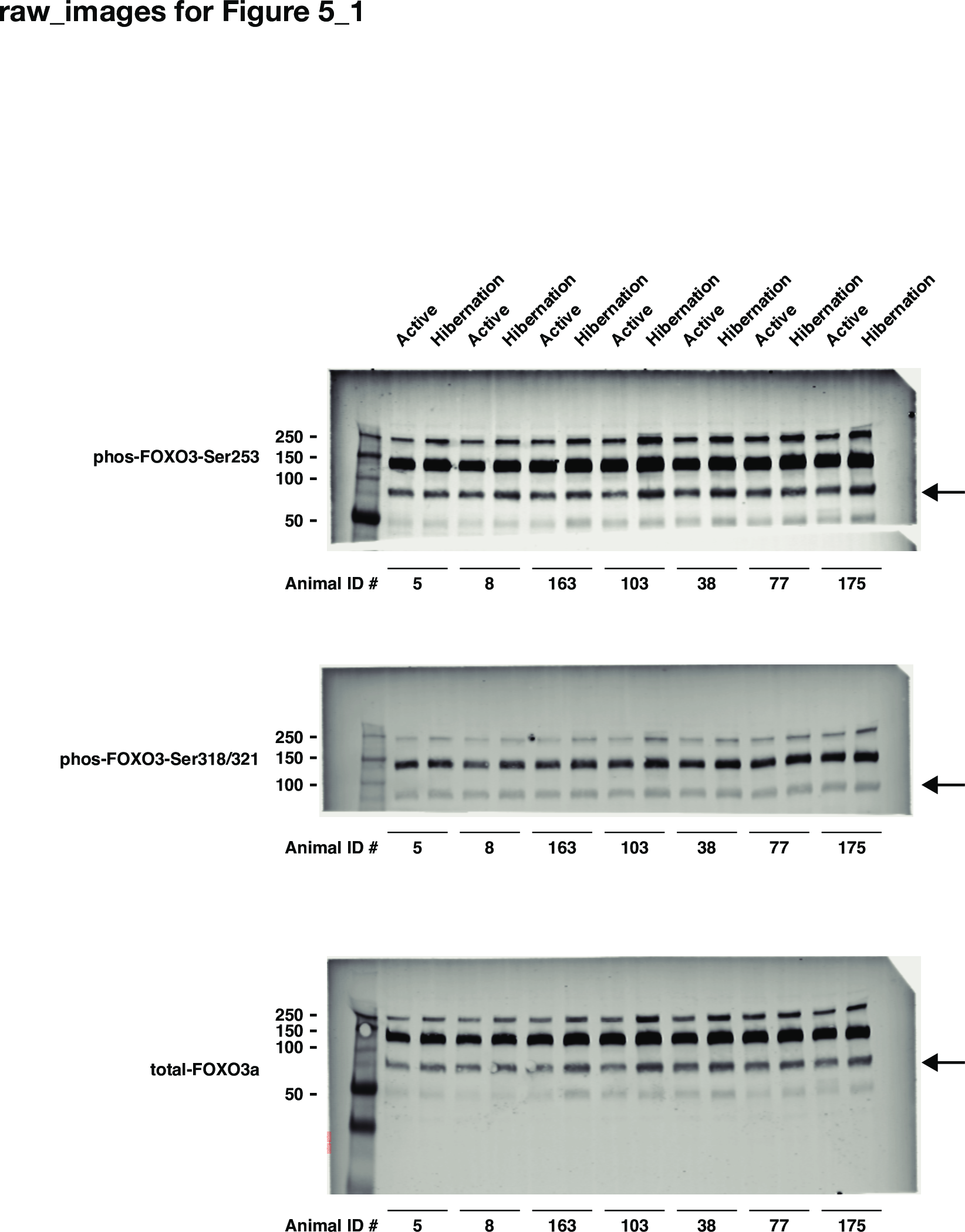

Supplement: S5 Raw images — (TIF) [file pone.0263085.s005.tif]

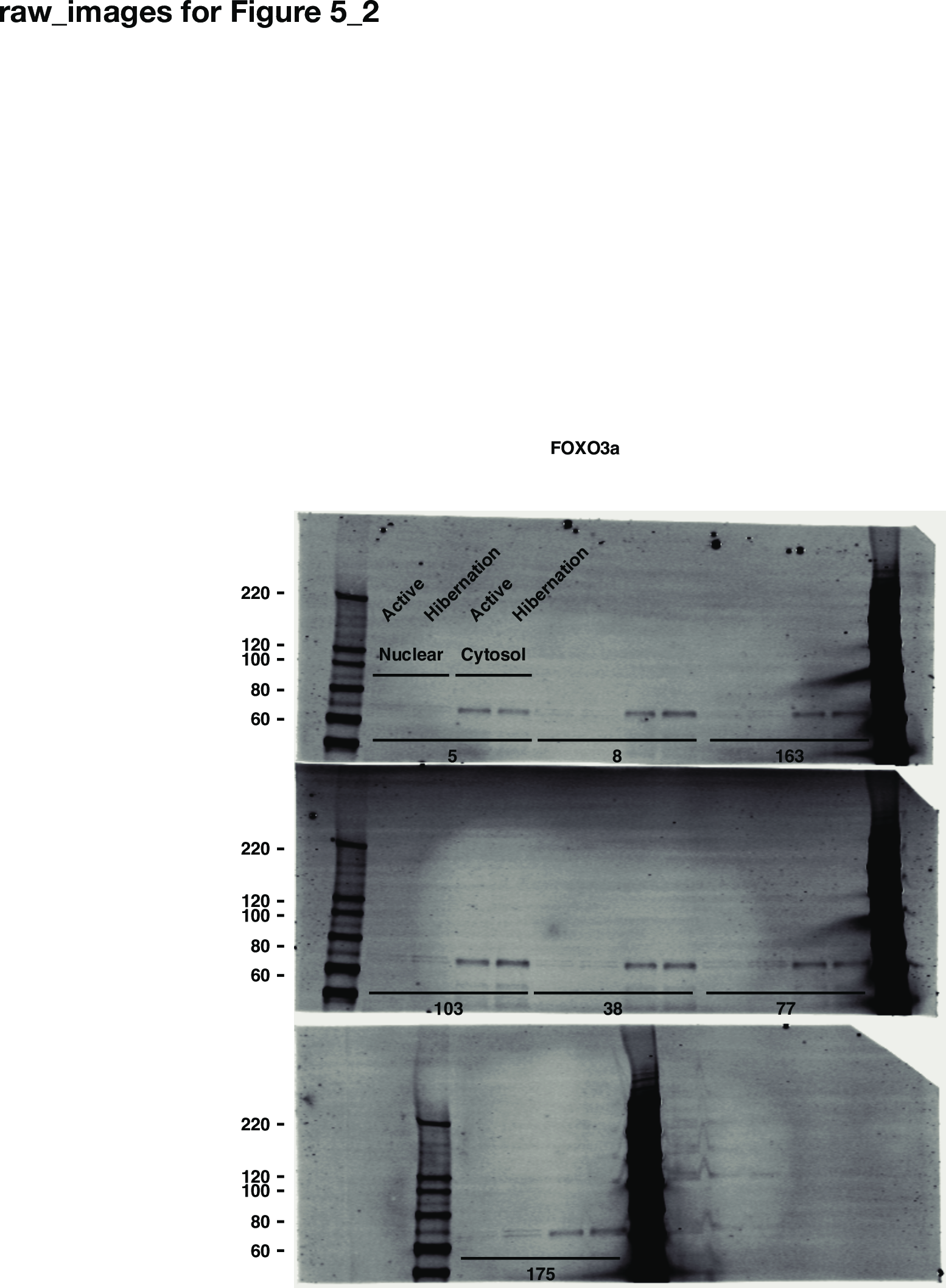

Supplement: S6 Raw images — (TIF) [file pone.0263085.s006.tif]

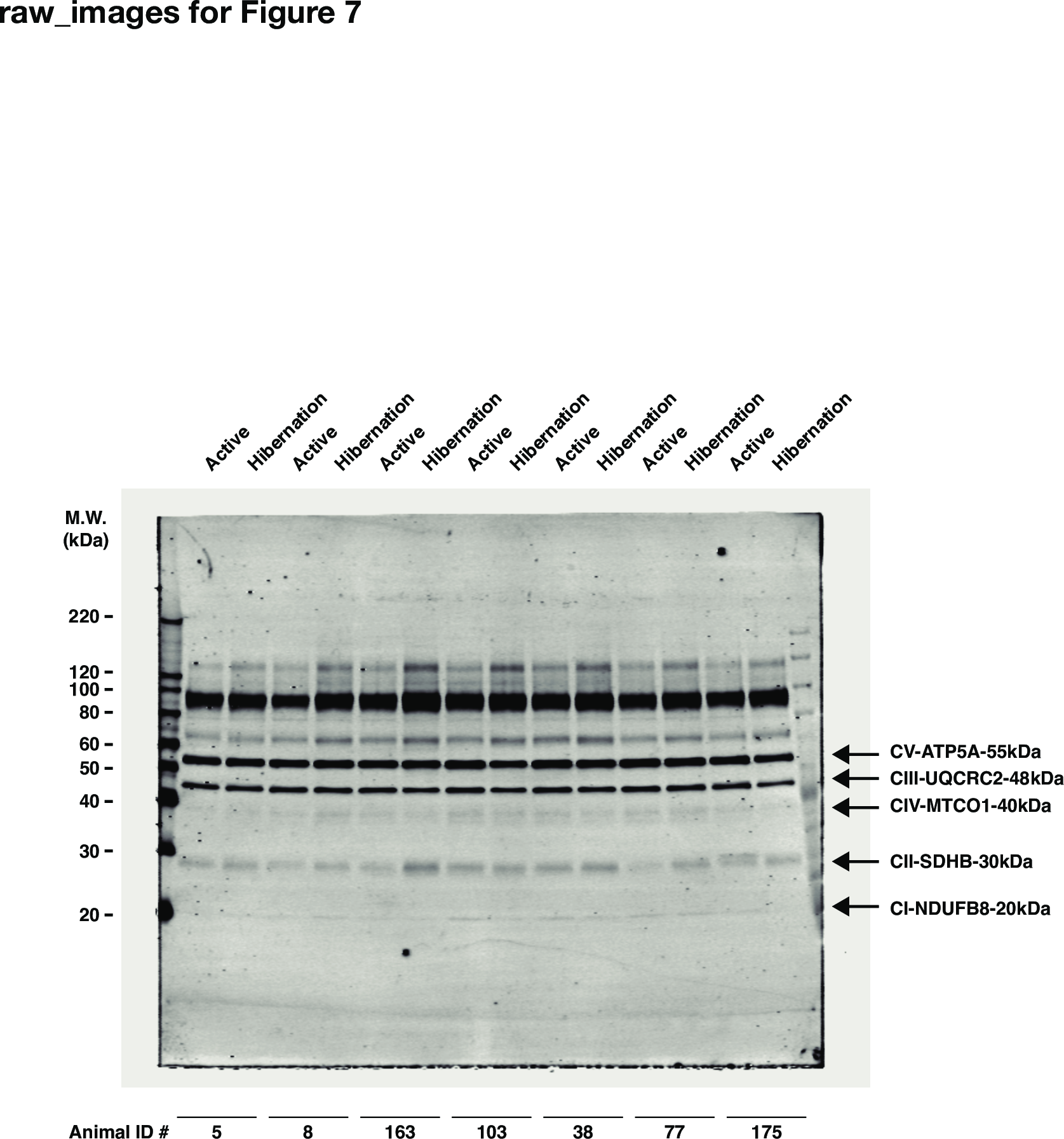

Supplement: S7 Raw images — (TIF) [file pone.0263085.s007.tif]
